# Anion Dependent Self-Assembly of Polynuclear Cd-Ln Schiff Base Nanoclusters: NIR Luminescent Sensing of Nitro Explosives

**DOI:** 10.3389/fchem.2019.00139

**Published:** 2019-03-20

**Authors:** Hongfen Chen, Xiaoping Yang, Weizhong Jiang, Dongmei Jiang, Dongliang Shi, Bichen Yuan, Fei Wang, Lijie Zhang, Shaoming Huang

**Affiliations:** ^1^Zhejiang Key Laboratory of Carbon Materials, College of Chemistry and Materials Engineering, Wenzhou University, Wenzhou, China; ^2^Guangzhou Sysmyk New Material Science & Technology Co., Ltd., Guangzhou, China

**Keywords:** self-assembly, schiff base ligand, nanoclusters, NIR lanthanide luminescence, sensing of nitro explosives

## Abstract

Two types of polynuclear Cd-Ln complexes [CdLnL(NO_3_)Cl_2_(DMF)_2_] [Ln = La (**1**) and Nd (**2**)] and [Ln_2_CdL_2_(NO_3_)_2_(DMF)_2_](OH)_2_ [Ln = La (**3**) and Nd (**4**)] were constructed using a new Schiff base ligand which has a long backbone with two phenyl groups. The Schiff base ligands show a “twist” configuration in **1**–**4**. The crystal structures show that the molecular dimensions of **3** and **4** are about 6 × 10 × 15 Å. The Cd-Nd complexes **2** and **4** exhibit the typical NIR luminescence of Nd^3+^. Interestingly, **4** shows the luminescent sensing of nitro explosives and exhibits a high sensitivity to 2-nitrophenol at the ppm level.

## Introduction

Currently, a great deal of attention is being paid to the lanthanide-based fluorescent chemosensors due to their unique optical properties (i.e., long lifetimes, line-like emission bands, and large Stokes' shifts; Jankolovits et al., 2011) and potential application in the detection of various analytes such as metal ions (Chen et al., 2009; Tang et al., 2013), anions (Qiu et al., 2009; Shi et al., 2015), and small molecules (Guo et al., 2011; Liu et al., 2013). Many visible luminescent Eu- and Tb-based frameworks such as Eu- and Tb-MOFs have been designed for this purpose (Chen et al., 2009; Qiu et al., 2009; Guo et al., 2011; Liu et al., 2013; Tang et al., 2013; Shi et al., 2015). In contrast, there are very few reports on the near-infrared (NIR) luminescent probes based on polynuclear lanthanide complexes, for example, Yb(III), Nd(III), and Er(III) complexes (Wu et al., 2018). In fact, NIR luminescent lanthanide complexes have been used as luminescent labels in the study of biological imaging and bioanalytical detection due to their low signal-to-noise ratios in living organisms (Hemmila and Webb, 1997; Stouwdam et al., 2003; Zheng et al., 2014).

**Scheme 1:**
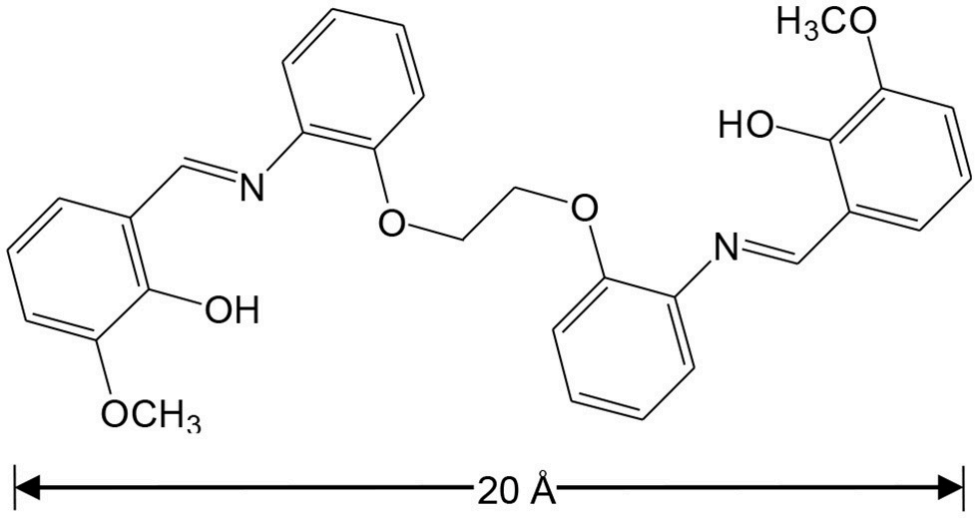
Schiff base ligand H_2_L.

It is known that light-absorbing Zn(II) and Cd(II) chromophores can be used as sensitizers for lanthanide emission in d-f complexes (“antenna effect”) (Zheng et al., 2004; Zhu et al., 2006). We recently reported our studies focused on sensing with NIR luminescent Zn-Ln and Cd-Ln clusters formed by flexible salen-type Schiff base ligands, with long carbon-carbon (-CH_2_-CH_2_-) backbones (Jiang et al., 2018; Wang et al., 2018). The backbones of Schiff base ligands can efficiently affect the structures of d-f complexes. Thus, we present the synthesis and the structural characterization of two types of Cd-Ln complexes, with a specially designed Schiff base ligand 6,6′-((1Z,1′E)-(((ethane-1,2-diylbis(oxy))bis(2,1-phenylene))bis(azanylylidene))bis(methanylylidene))bis(2-methoxyphenol) (H_2_L), which has a long backbone with two phenyl groups (Scheme 1). These new complexes are [CdLnL(NO_3_)Cl_2_(DMF)_2_] [Ln = La (**1**) and Nd (**2**)] and [Ln_2_CdL_2_(NO_3_)_2_(DMF)_2_](OH)_2_ [Ln = La (**3**) and Nd (**4**)]. The length of H_2_L is approximately 20 Å, which helps to form large metal complexes. For example, molecules **3** and **4** are of nanoscale proportions, with the molecular dimensions ~6 × 10 × 15 Å. The Schiff base ligand H_2_L has four phenyl groups, which is advantageous to the formation of π···π electrostatic interactions with added explosives. Of particular note, **4** shows NIR luminescent sensing of nitro explosives, and exhibits high sensitivity to 2-nitrophenol (2-NP).

## Experimental Section

### Materials and Methods

Metal salts and solvents were purchased from Meryer and used directly without further purification. All reactions were performed in dry oxygen-free dinitrogen atmospheres using standard Schlenk techniques. Physical measurements: Powder XRD: D8ADVANCE; IR: Nicolet IS10 spectrometer. Melting points were obtained in sealed glass capillaries under dinitrogen and were uncorrected. Elemental analyses (C, H, N) were carried out on a EURO EA3000 elemental analysis. The thermogravimetric analyses were carried out on a TA Instruments Q600 under flowing N_2_ (200.0 mL/min) with a heating rate of 10.00°C/min from ambient temperature to 900°C. Field emission scanning electron microscopy (FESEM) images and EDX spectra were recorded on a Nova NanoSEM 200 scanning electron microscope.

### Preparation of the Schiff Base Ligand H_2_L

2-[2-(2-aminophenoxy)ethoxy]phenylamine (2.80 mmol, 0.6840 g) in 20 mL EtOH was added drop by drop under reflux, to a solution of 2-hydroxy-3-methoxybenzaldehyde (5.60 mmol, 0.8520 g) in 10 mL EtOH. The yellow solution was then stirred for 3.5 h under reflux. The resulting yellow solid was filtered off, washed with 5 mL EtOH three times, and air dried. Yield (based on 2-[2-(2-Aminophenoxy)ethoxy]phenylamine): 1.3911 (97%). m.p. = 194.2°C. Elemental analysis: Found: C, 70.45; H, 5.62; N, 5.58%; Calc. for C_30_H_28_N_2_O_6_: C, 70.30; H, 5.51; N, 5.47%. IR (cm^−1^): 1,606 (m), 1,470 (w), 1,348 (w), 1,268 (w), 1,119 (m), 1,059 (m), 950 (w), 855 (m), 799 (w), 746 (s), 673 (s), 658 (s). ^1^H NMR (DMSO, 500 MHz): δ 13.94 (s, 2H), 8.93 (s, 2H), 7.41 (d, 2H), 7.24 (t, 4H), 7.05 (dd, 6H), 6.80 (t, 2H), 4.25 (s, 4H), 3.79 (d, 6H).

### Preparation of [CdLaL(NO_3_)Cl_2_(DMF)_2_] (1)

CdCl_2_ (0.2 mmol, 0.0367 g), La(NO_3_)_3_·6H_2_O (0.2 mmol, 0.0650 g) and H_2_L (0.2 mmol, 0.1024 g) were dissolved in 5 mL MeOH, 5 mL EtOH and 2 mL DMF at room temperature, respectively, and then mixed together. A solution of NEt_3_ in EtOH (0.35 mol/L, 1 mL) was added into the mixture. The yellow solution was stirred for 30 min under reflux and then filtered. The filtrate was transferred into a test tube, and then the test tube was placed in a jar with diethyl ether. The diethyl ether diffused slowly into the filtrate to create a pale yellow crystalline solid. The crystalline product was filtered off and air dried. Yield (based on La(NO_3_)_3_·6H_2_O): 0.0750 (36%). m.p. > 150.4°C (dec.) Elemental analysis: Found: C, 41.58; H, 3.91; N, 6.79 %. Calc. for LaCdCl_2_C_36_H_40_O_11_N_5_: C, 41.50; H, 3.84; N, 6.72 %. MS(ESI): 399.2294 (M+H)^+^, 513 ([H_2_L+H]^+^), 625 ([CdL+H]^+^), 648 ([LaL]^+^), 771 ([Cd_2_LCl]^+^), 1005 ([M-Cl^−^]^+^). IR (cm^−1^): 1,648 (m), 1,530 (m), 1,384 (w), 1,258 (w), 1,079 (m), 1,009 (m), 917 (m), 839 (m), 738 (s), 678 (s).

### [CdNdL(NO_3_)Cl_2_(DMF)_2_] (2)

The pale-yellow crystalline product of this complex was obtained using Nd(NO_3_)_3_·6H_2_O (0.2 mmol, 0.0661 g) by a similar method described for **1**. Yield (based on Nd(NO_3_)_3_·6H_2_O): 0.0711 (34%). m. p. > 133.8°C (dec.). Elemental analysis: Found: C, 41.50; H, 3.89; N, 6.82 %. Calc. for NdCdCl_2_C_36_H_40_O_11_N_5_: C, 41.30; H, 3.82; N, 6.69 %. IR (cm^−1^): 1,636 (m), 1,530 (m), 1,401 (w), 1,275 (w), 1,179 (w), 1,053 (m), 991 (w), 882 (m), 809 (m), 738 (m), 670 (s).

### Preparation of [La_2_CdL_2_(NO_3_)_2_(DMF)_2_](OH)_2_ (3)

Cd(NO_3_)_2_·4H_2_O (0.2 mmol, 0.0617 g), La(NO_3_)_3_·6H_2_O (0.2 mmol, 0.0650 g) and H_2_L (0.2 mmol, 0.1,024 g) were dissolved in 5 mL MeOH, 5 mL EtOH and 5 mL DMF at room temperature, respectively, and then mixed together. A solution of NEt_3_ in EtOH (0.35 mol/L, 3 mL) was added into the mixture. The resulting yellow solution was processed in the same way described for **1** to obtain a pale yellow crystalline product of this complex. Yield (based on La(NO_3_)_3_·6H_2_O): 0.1225 (33%). m. p. > 245.8°C (dec.). Elemental analysis: Found: C, 45.35; H, 4.55; N, 6.83%. Calc. for La_2_CdC_70_H_83_O_26_N_9_: C, 45.26; H, 4.47; N, 6.79%. IR (cm^−1^): 1,633 (w), 1,540 (m), 1,377 (m), 1,232 (w), 1,062 (m), 968 (w), 849 (w), 758 (s), 666 (s).

### Preparation of [Nd_2_CdL_2_(NO_3_)_2_(DMF)_2_](OH)_2_ (4)

The pale-yellow crystalline product of this complex was obtained using Nd(NO_3_)_3_·6H_2_O (0.2 mmol, 0.0661 g) using a similar method described for **3**. Yield (based on Nd(NO_3_)_3_·6H_2_O): 0.1307 (35%). m. p. > 246.6 °C (dec.). Elemental analysis: Found: C, 45.10; H, 4.53; N, 6.80 % Calc. for Nd_2_CdC_70_H_83_O_26_N_9_: C, 44.99; H, 4.45; N, 6.75 %. IR (cm^−1^): 1,596 (m), 1,500 (m), 1,384 (m), 1,291 (w), 1,132 (m), 1,062 (m), 951 (w), 867 (s), 787 (m), 646 (s).

### Crystallography

The diffraction experiments were carried out on a Smart APEX CCD diffractometer in the θ−2θ mode with monochromated Mo-Kα radiation (λ = 0.71073 Å). The structures were solved by direct methods (SHELX 97 program) (Sheldrick, 1997). All non-hydrogen atomic coordinates were refined anisotropically. Hydrogen atoms at their calculated positions were included in the structure factor calculation but were not refined. Selected bond lengths (Å) and angles (°) in the structures of **1**–**4** are shown in Tables S1–S4 (ESI). The CCDC reference numbers for the crystal structures are 1,865,266–1,865,269, respectively.

For **1**: C_36_H_40_N_5_O_11_Cl_2_CdLa, monoclinic, space group P2(1)/n, *a* = 12.177(5), *b* = 19.331(7), *c* = 19.141(8) Å, α = 90°, β = 96.738(7)°, γ = 90°, *V* = 4,475(3) Å^3^, *Z* = 4, *Dc* = 1.545 g cm^−3^, μ(Mo-Kα) = 1.594 mm^−1^, *F*_(000)_ = 2,072, *T* = 190 K. *R*_1_ = 0.1013, *wR*_2_ = 0.2029, GOF = 1.115.

For **2**: C_36_H_40_N_5_O_11_Cl_2_CdNd, monoclinic, space group P2(1)/n, *a* = 11.800(5), *b* = 17.258(7), *c* = 19.859(8) Å, α = 90°, β = 93.755(8)°, γ = 90°, *V* = 4,035(3) Å^3^, *Z* = 4, *Dc* = 1.722 g cm^−3^, μ(Mo-Kα) = 1.995 mm^−1^, *F*_(000)_ = 2084, *T* = 190 K. *R*_1_ = 0.0554, *wR*_2_ = 0.1765, GOF = 1.016.

For **3**: C_70_H_83_N_9_O_26_CdLa_2_, orthorhombic, space group Pbcn, *a* = 32.423(10), *b* = 30.831(10), *c* = 15.887(5) Å, α = 90°, β = 90°, γ = 90°, *V* = 15,882(8) Å^3^, *Z* = 8, *Dc* = 1.547 g cm^−3^, μ(Mo-Kα) = 1.403 mm^−1^, *F*_(000)_ = 7,432, *T* = 190 K. *R*_1_ = 0.0996, *wR*_2_ = 0.2557, GOF = 1.178.

For **4**: C_70_H_83_N_9_O_26_CdNd_2_, orthorhombic, space group Pbcn, *a* = 32.458(11), *b* = 30.698(9), *c* = 15.924(6) Å, α = 90°, β = 90°, γ = 90°, *V* = 15,866(9) Å^3^, *Z* = 8, *Dc* = 1.558 g cm^−3^, μ(Mo-Kα) = 1.636 mm^−1^, *F*_(000)_ = 7,480, *T* = 190 K. *R*_1_ = 0.0858, *wR*_2_ = 0.2489, GOF = 1.075.

### Photophysical Studies

The UV-visible absorption spectra were recorded at RT using an UV-3600 spectrophotometer. The solvent employed was of HPLC grade. Luminescence spectra in the visible and NIR regions were recorded on a FLS 980 fluorimeter. The light source for excitation and emission spectra was a 450 W xenon arc lamp with a continuous spectral distribution from 190 to 2,600 nm. A liquid nitrogen cooled Ge PIN diode detector was used to detect the NIR emissions from 800 nm to 1,700 nm. The temporal decay curves of the fluorescence signals were stored using the attached storage digital oscilloscope. The overall emission quantum yields (Φ_em_) were obtained using an integrating sphere, according to Equation Φ_em_ = *N*_em_/*N*_abs_, where *N*_em_ and *N*_abs_ are the numbers of emitted and absorbed photons, respectively. The intrinsic quantum yields (Φ_Ln_) of Ln^3+^ emission is calculated using Φ_Ln_ = τ/τ_0_, where τ and τ_0_ are the observed emission lifetime and the natural lifetime of Ln^3+^, respectively. Systematic errors were deducted through the standard instrument corrections. All measurements were carried out at room temperature. For the luminescent response experiment, the lanthanide NIR emissions of **4** were recorded when various concentrations of explosives were added into the solution of the complex with the initial concentration of 15 μM.

## Results and Discussion

### Synthesis and Structures

**Figure 1:**
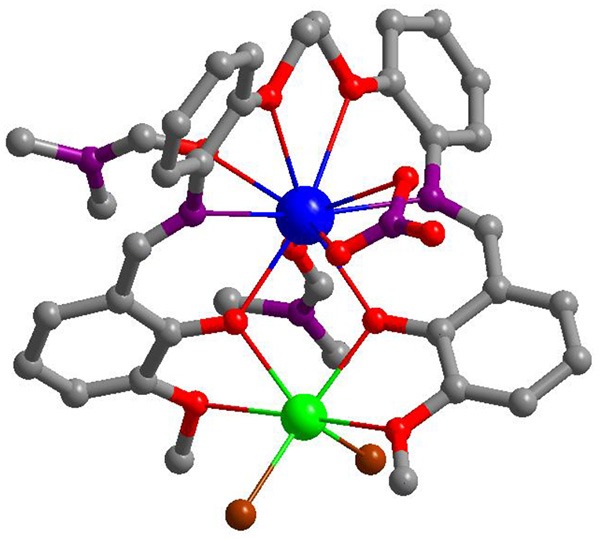
A view of the crystal structure of **2**. (Nd^3+^: blue; Cd^2+^: green; Cl: brown; N: purple; O: red; C: gray).

The synthesis of the new Schiff base ligand H_2_L was accomplished using preparations from literature (Lam et al., 1996), with a yield of 97% (Figure S1, Supporting Information) . The Cd-Ln complexes were synthesized from the reactions of H_2_L with CdCl_2_ and Ln(NO_3_)_3_·6H_2_O (Ln = La and Nd). The isomorphous **1** and **2** were obtained as pale-yellow crystalline solids. As shown in Figure 1, in **2** the Nd^3+^ and Cd^2+^ ions were bridged by two phenolic oxygen atoms of the Schiff base ligand with a separation of 3.872 Å. The coordination number of Nd^3+^ ion is ten, coordinated with eight O atoms from one L ligand, one ${\text{NO}}_{3}^{-}$ anion and two DMF molecules and two N atoms from the L ligand. The Cd^2+^ ion is surrounded by four oxygen atoms from the L ligand and two Cl^−^ anions. The Schiff base ligand coordinated with both metal ions through its two N and six O atoms. The bond lengths of Cd-O, Nd-N and Nd-O in **2** are 2.284–2.595, 2.758–2.783, and 2.424–2.740 Å, respectively.

**Figure 2:**
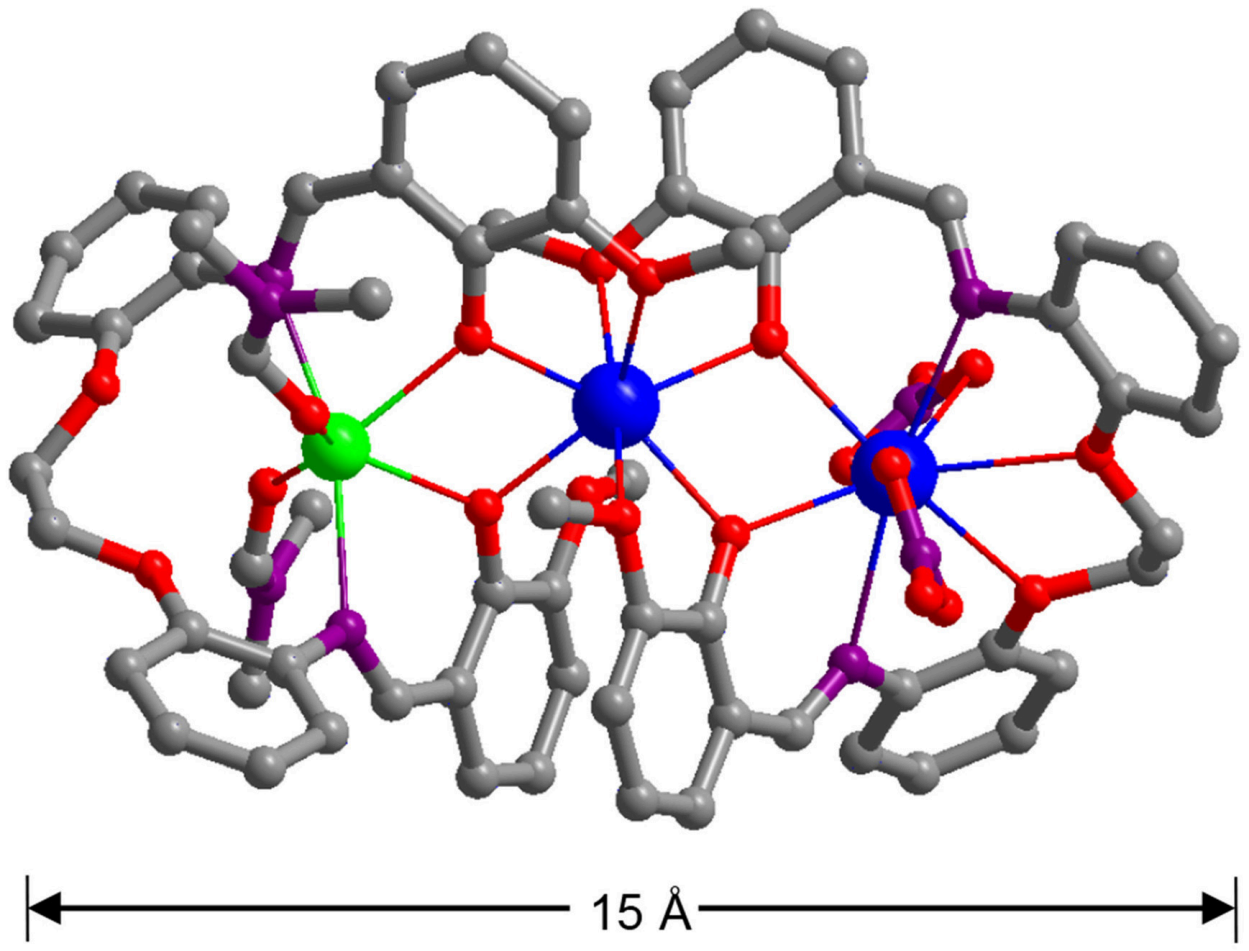
A view of the crystal structure of **4**. (Nd^3+^: blue; Cd^2+^: green; N: purple; O: red; C: gray).

The nature of anions that existed in the reactions appears to have affected the self-assembly process of the clusters. Thus, the reactions of H_2_L with Cd(NO_3_)_2_·4H_2_O and Ln(NO_3_)_3_·6H_2_O (Ln = La and Nd) under similar experimental conditions produced isomorphous **3** and **4**. The crystal structure of **4** is shown in Figure 2. Two Nd^3+^ and one Cd^2+^ ions are coordinated with two Schiff base ligands. The outer Nd^3+^ ion is bound by the O_2_N_2_O_2_ core of one L ligand in addition to four O atoms from two ${\text{NO}}_{3}^{-}$ anions, resulting in a ten-coordinate geometry. While the center Nd^3+^ ion is eight-coordinates and bound by the O_2_O_2_ cavities of two L ligands. The Cd^2+^ ion is surrounded by four O atoms from the L ligand and two DMF molecules and two N atoms from the L ligand. The center Nd^3+^ ion is bridged with the outer Nd^3+^ and Cd^2+^ ions through four phenolic oxygen atoms of the Schiff base ligands. The Nd-Nd and Nd-Cd distances are 3.823 and 3.719 Å, respectively. In **4**, the bond lengths of Cd-O, Nd-N, and Nd-O are 2.262–2.322, 2.699–2.950, and 2.218–2.888 Å, respectively.

**Figure 3:**
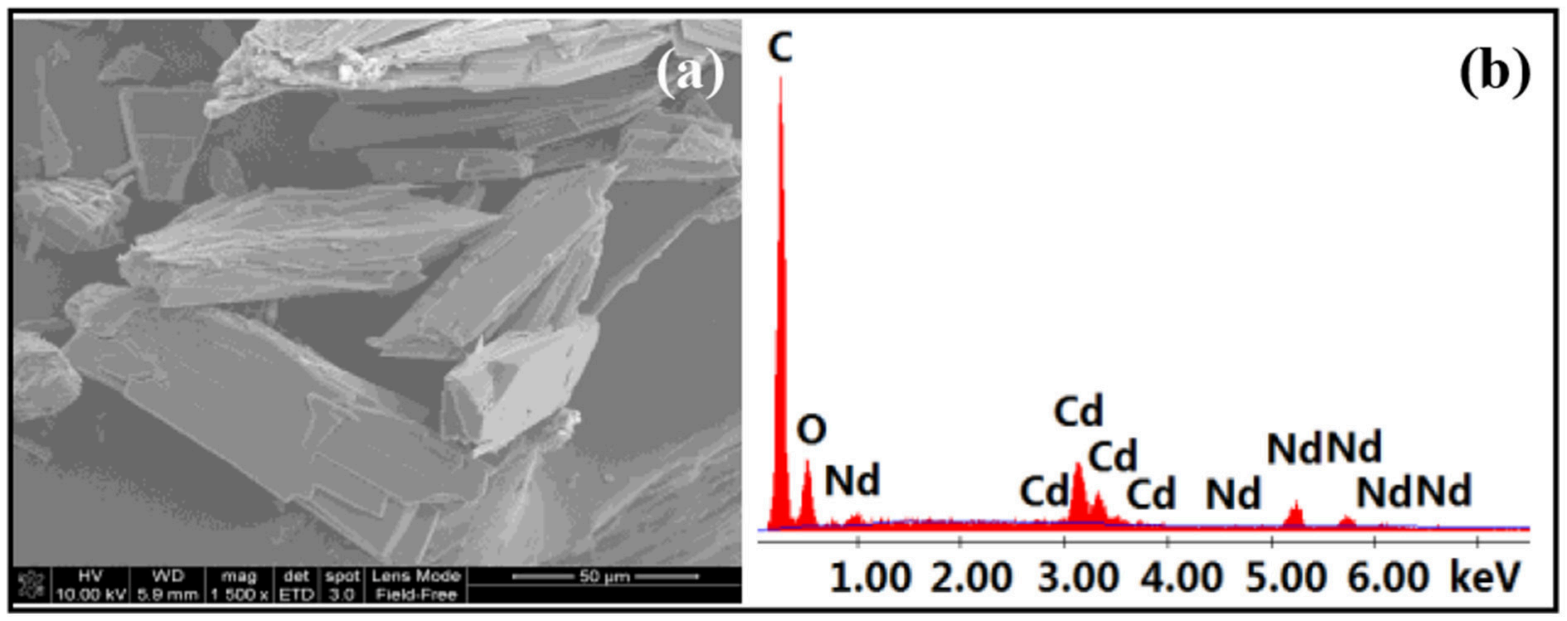
Scanning electron microscopy image **(a)** and energy dispersive X-ray spectroscopy spectrum **(b)** of **4**.

The long Schiff base ligands show a “twist” configuration in **1**–**4**, resulting in large molecular dimensions of the complexes. For example, the molecular sizes of **3** and **4** are about 6 × 10 × 15 Å. The panoramic scanning electron microscopy (SEM) image and energy dispersive X-ray spectroscopy (EDX) spectrum of **4** are shown in Figure 3. The molar ratio of Cd:Nd in **4** is confirmed to be 1:2 (Figure 3b), which is consonant with the crystal structure. The powder XRD patterns of the **1** and **4** show large background peaks, indicating that they are predominantly amorphous (Figure S2, Supporting Information). Thermogravimetric analyses show that **1**–**4** lose about 2% of the weight before 100°C (Figure S3, Supporting Information), due to the escape of uncoordinated solvent molecules such as MeOH and H_2_O.

Melting point measurements indicate that **1–4** begin to discompose from 133 to 246°C (Experimental Section). Besides the molecular ion peak (m/z = 1,005), the mass spectrum of **1** shows fragments of the free ligand [H_2_L+H]^+^, [CdL+H]^+^, [LaL]^+^, [Cd_2_LCl]^+^ at 513, 625, 648, and 771, respectively (Figure S4, Supporting Information). This indicates that besides the product of 1, other species such as Cd-L, La-L, or Cd-Cd-L complexes may exist in the solution after the reaction. The products of **1–4** were collected form their solutions as crystalline solids.

### Photophysical Properties

**Figure 4:**
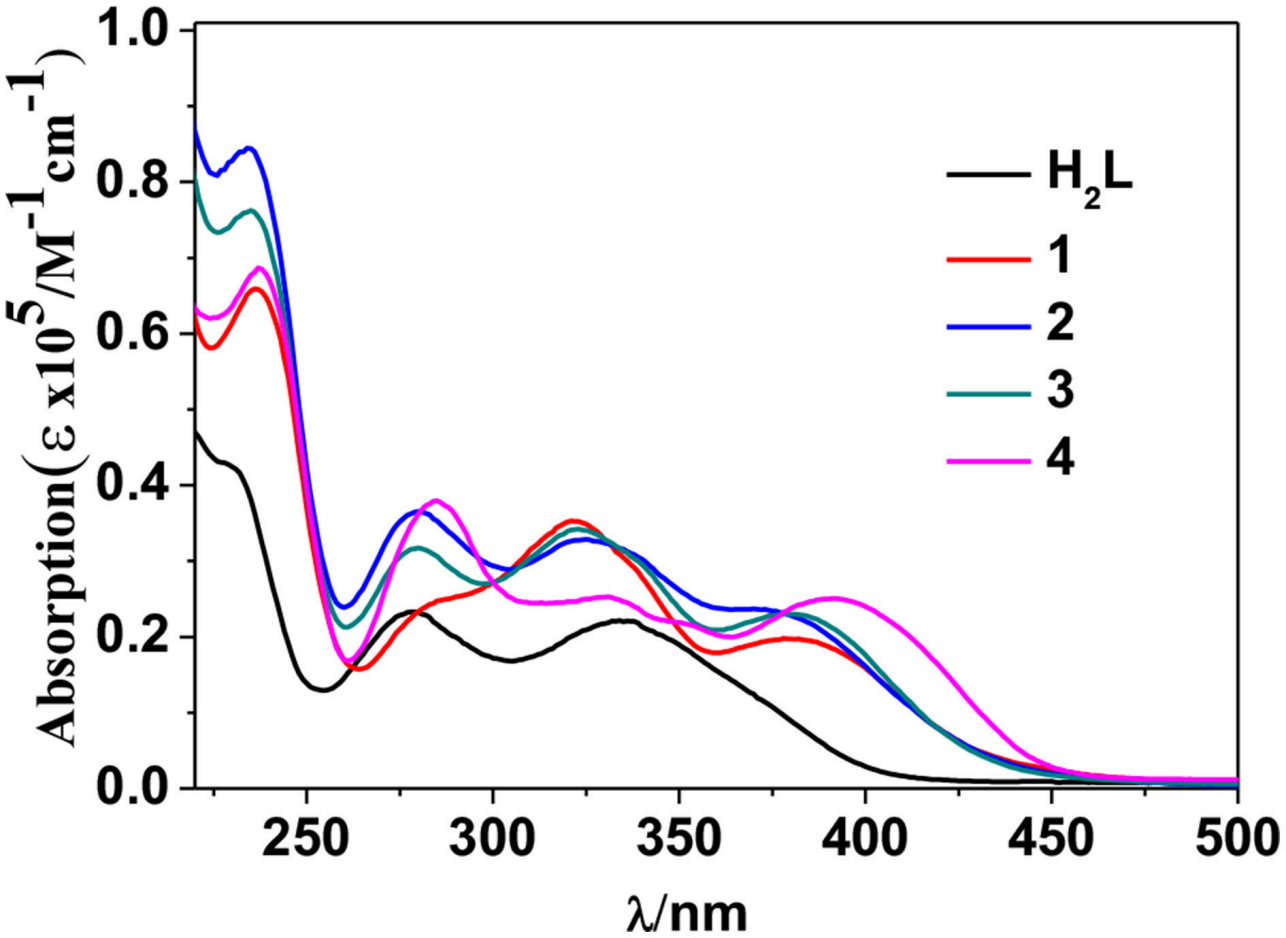
UV-Vis spectra of the free ligand H_2_L and **1**–**4** in CH_3_CN. (C = 10^−6^-10^−5^ M).

**Figure 5:**
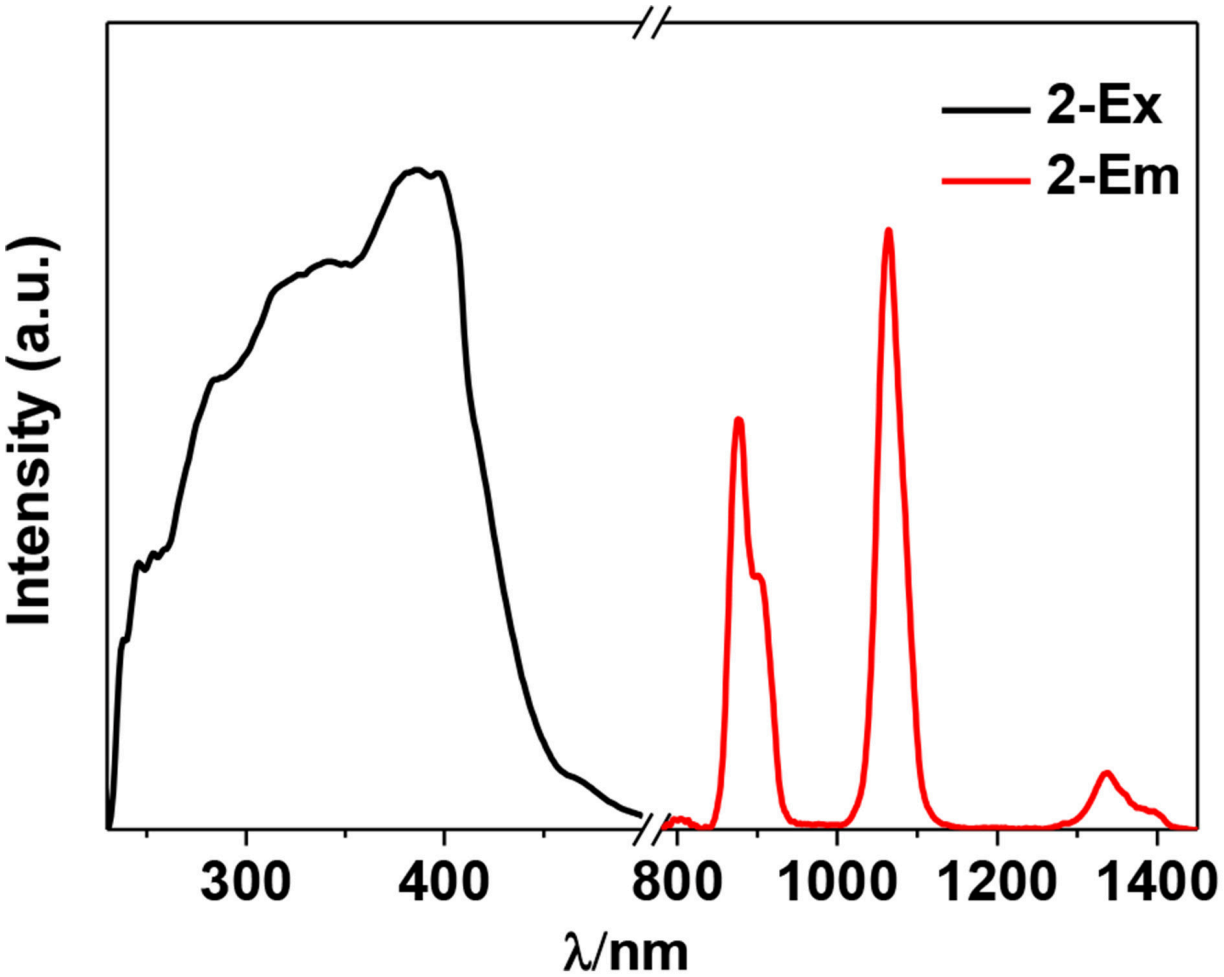
NIR luminescence spectra of **2** in CH_3_CN. (λ_em_ = 1,064 nm, λ_ex_ = 388 nm).

**Figure 6:**
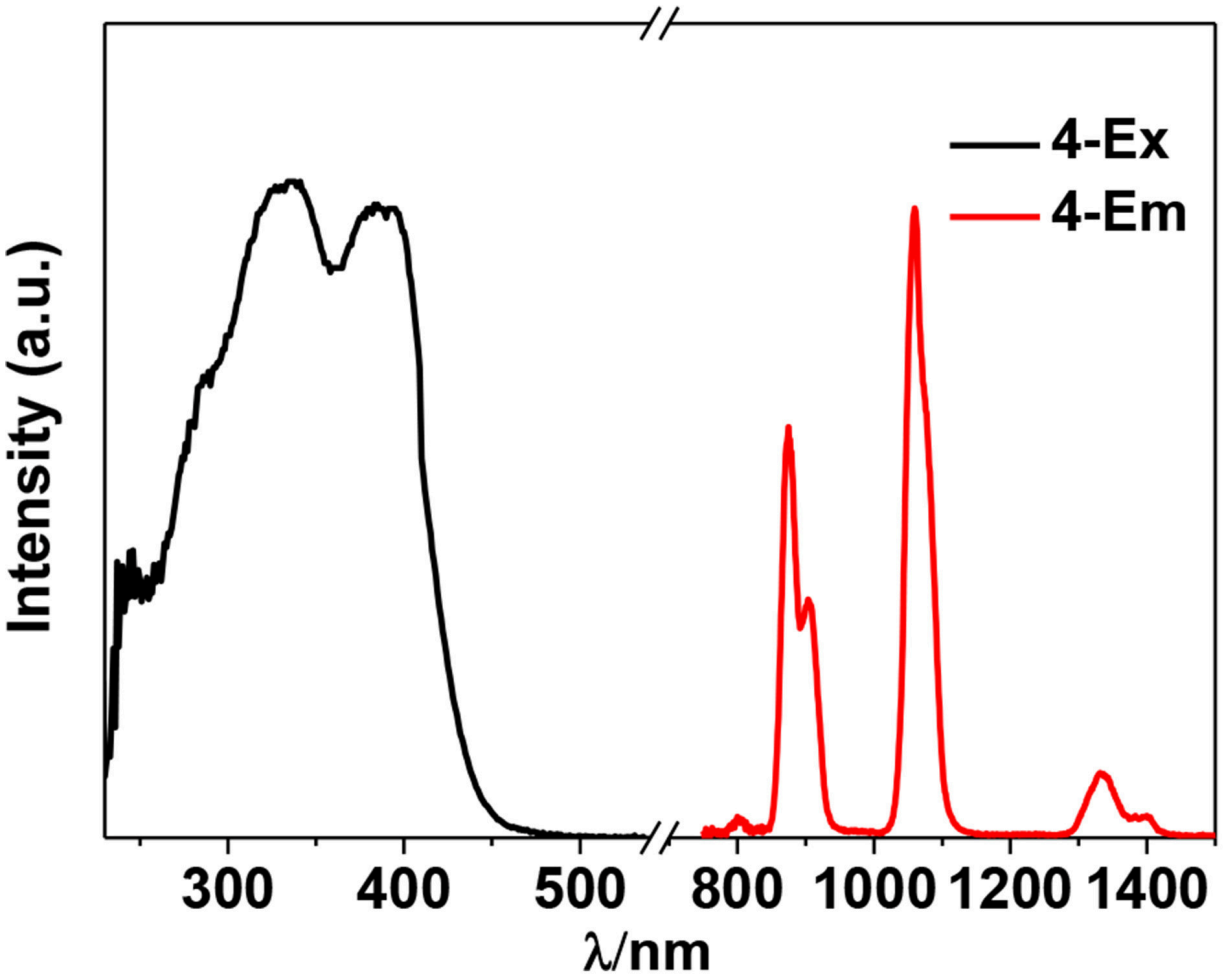
NIR luminescence spectra of **2** in CH_3_CN. (λ_em_ = 1,060 nm, λ_ex_ = 386 nm).

The photophysical properties of **1**–**4** were studied in solution. The UV-vis absorption spectra of the free Schiff base ligand and **1**–**4** are shown in Figure 4. Compared to the absorption bands of the free ligand H_2_L, some of **1**–**4** are red-shifted. It is noticeable that, a broad absorption band at about 400 nm was found for **1**–**4**, which may be from the ligand-to-metal charge transfer (LMCT) transition due to the existence of Cd(II) ions in the complexes (Blasse, 1994). For the Cd-La complexes **1** and **3**, excitations of the ligand-centered absorption bands result in broad visible ligand-centered ^1^π-π^*^ emission bands at 548 and 554 nm, respectively (Figure S5, Supporting Information), which are blue-shifted compared to that of the free ligand H_2_L (λ_max_ = 602 nm). While, for the Cd-Nd complexes **2** and **4**, besides the visible ligand-centered emission bands, they also show NIR luminescence of Nd^3+^ (^4^F_3/2_→^4^I_j/2_ transitions, *j* = 9, 11, and 13) (Figures 5, 6). For the NIR luminescence, both **2** and **4** show broad excitation bands (i.e., λ_ex_ = 327 and 386 nm for **4**), indicating that the chromogenic Cd/L moieties can act as effective sensors for the luminescence of Nd^3+^ ions (Sabbatini et al., 1993; María et al., 2017). The excitation and emission wavelengths (λ_ex_ and λ_em_) as well as the absorption of excitation wavelengths (ε), luminescence lifetimes (τ) and overall luminescence quantum yields (Φ_em_) of **2** and **4** in solution are listed in Table 1.

**Table 1:** The excitation and emission wavelengths (λ_ex_ and λ_em_), the absorption of excitation wavelengths (ε), lifetimes (τ), and quantum yields (Φ_em_) of **1**–**4** in solution.

**Clusters**	**λ_ex_ (nm)/ε (×10^**5**^ M^**−1**^cm^**−1**^)**	**λ_em_ (nm)**	**τ (μs) (NIR/Vis)**	**Φ_em_ (%) (NIR/Vis)**
**1**	265/0.16, 411/0.11	548	—/10.18	—/7.16
**2**	388/0.21	556, 879, 1,064, 1,338	8.21/7.15	0.39/6.75
**3**	228/0.74, 334/0.32, 384/0.23	554	—/10.25	—/15.48
**4**	327/0.25, 386/0.25	559, 875, 1,060, 1,331	7.81(6.42^*a*^)/6.37	0.78(0.41^*a*^)/12.20

a *The addition of 400 μM 2-NP*.

**2** and **4** show typical NIR emission bands of Nd^3+^ from 875 to 1,338 nm (Table 1). The luminescence lifetimes (τ) of **2** and **4** in CH_3_CN are 8.21 μs and 7.81 μs, respectively (Figure S6, Supporting Information). Therefore, the intrinsic quantum yields (Φ_Ln_) of Nd^3+^ in **2** and **4** can be estimated at τ/τ_0_ = 3.28 and 3.12%, respectively, where τ_0_ = 250 μs [the natural lifetime of Nd^3+^ (Klink et al., 2000)]. As shown in Table 1, the overall NIR luminescence quantum yields (Φ_em_) of **2** and **4** are 0.39 and 0.78%, respectively, indicating that **4** shows better luminescence properties than **2**. This may be due to their different conformations and cooperative effects. For example, **4** has one more Schiff base ligand than **2** and can absorb and transfer more energy to the lanthanide ions. The efficiency (η_sens_) of the energy transfer from ligand to Ln^3+^ can be calculated from η_sens_ = Φ_em_/Φ_Ln_ (Bünzli and Piguet, 2005). Thus, the η_sens_ values in **2** and **4** are estimated to be 11.89 and 25.0%, respectively. For **1** and **3**, the La^3+^ ion does not have f-f transition energy levels, and therefore cannot accept any energy from the sensitizer. As shown in Table 1, the ligand-centered emission quantum yields of **1** and **3** in visible range are 7.16 and 15.48%, which are higher than those of **2** and **4**, respectively, due to no energy transfer to La^3+^ ion.

### Luminescent Sensing of Explosives

**Scheme 2:**
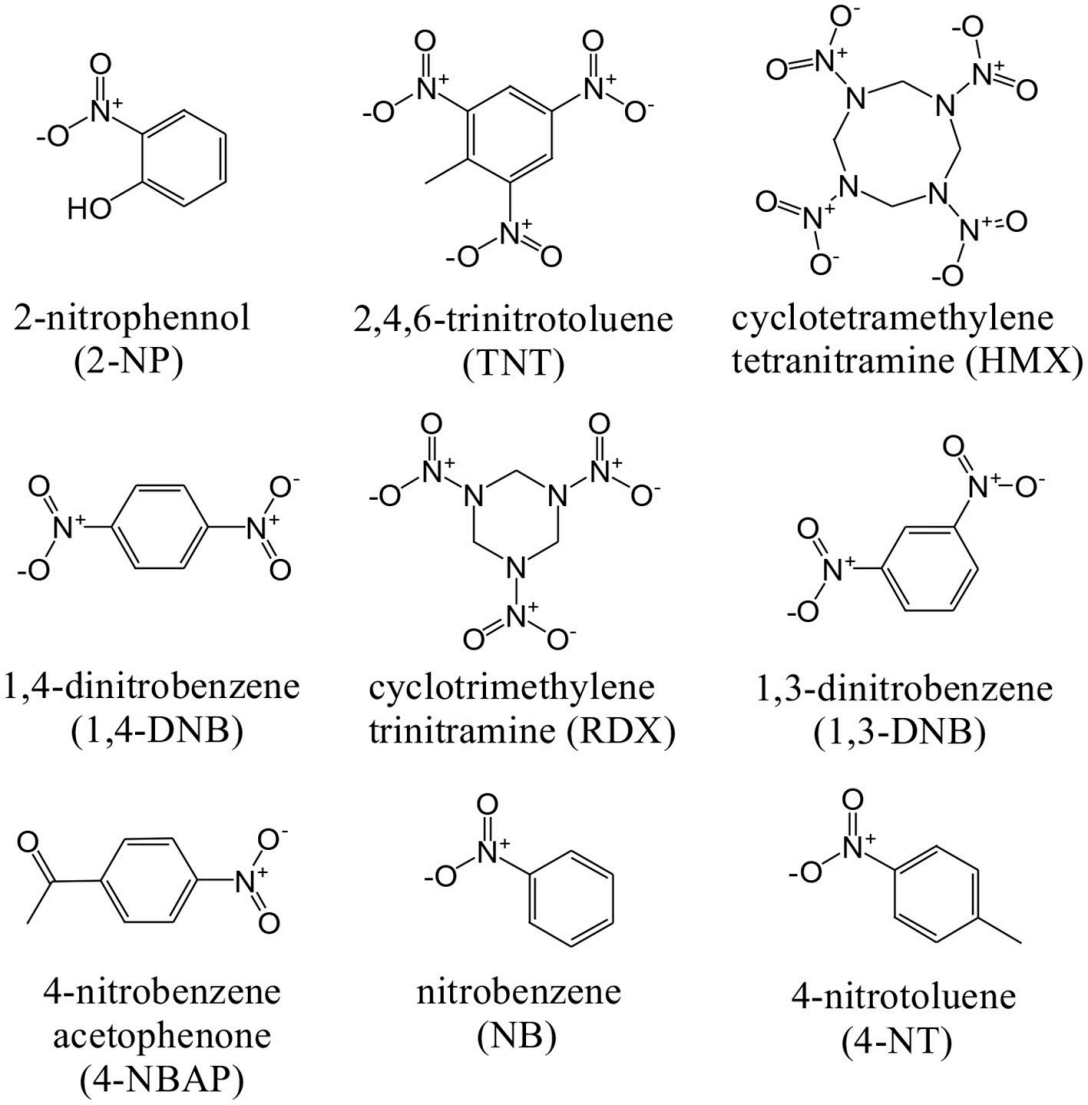
The structures of nitro explosives.

**Figure 7:**
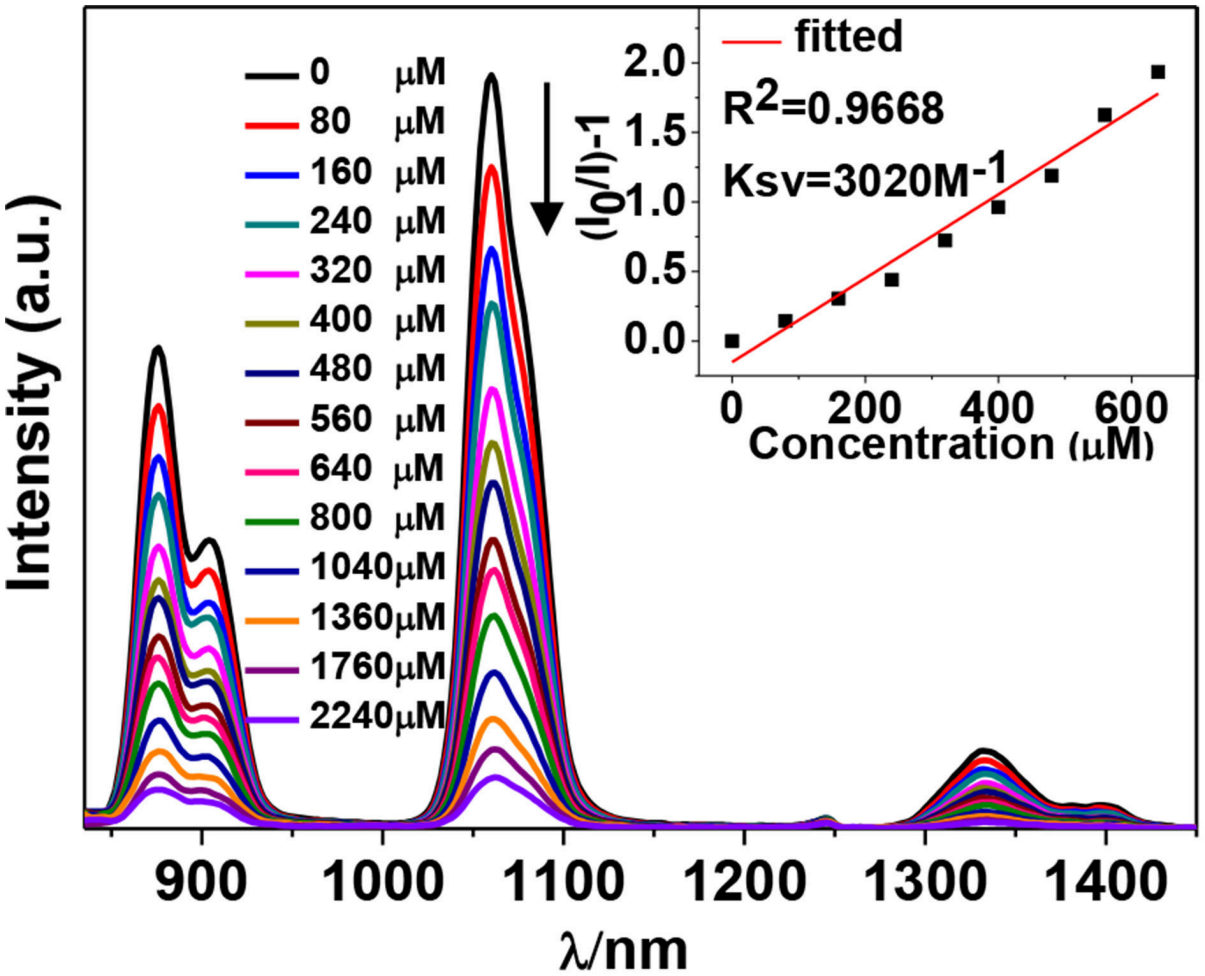
Decrease in the luminescence intensity of **4** (15 μM) in CH_3_CN upon the addition of different concentrations of 2-NP. Inset: linear relationship between the luminescence intensity and the concentration of 2-NP.

The NIR luminescent complex **4** has a larger surface area and more phenyl groups than **2**, which is favorable to the formation of intermolecular interactions between **4** and guest molecules. Thus, the NIR luminescent response of **4** to nitro explosives such as 2-nitrophenol (2-NP), 2,4,6-trinitrotoluene (TNT), cyclotetramethylene tetranitramine (HMX), 1,4- dinitrobenzene (1,4-DNB), cyclotrimethylene trinitramine (RDX), 1,3-dinitrobenzene (1,3-DNB), 4-nitrobenzene acetophenone (4-NBAP), nitrobenzene (NB) and 4-nitrotoluene (4-NT) has been studied in CH_3_CN (Scheme 2). The intensity of the strongest emission peak of **4** at 1,060 nm was recorded with the addition of the explosives. Interestingly, the NIR luminescence intensities of **4** are gradually decreased with the addition of explosives increase (Figure S7, Supporting Information). It is noticeable that the addition of 2-NP leads to much more rapid luminescence quenching than other explosives (Figure 7). For example, the addition of 400 μM 2-NP solution makes the emission intensity at 1,060 nm decrease about 50%.

**Figure 8:**
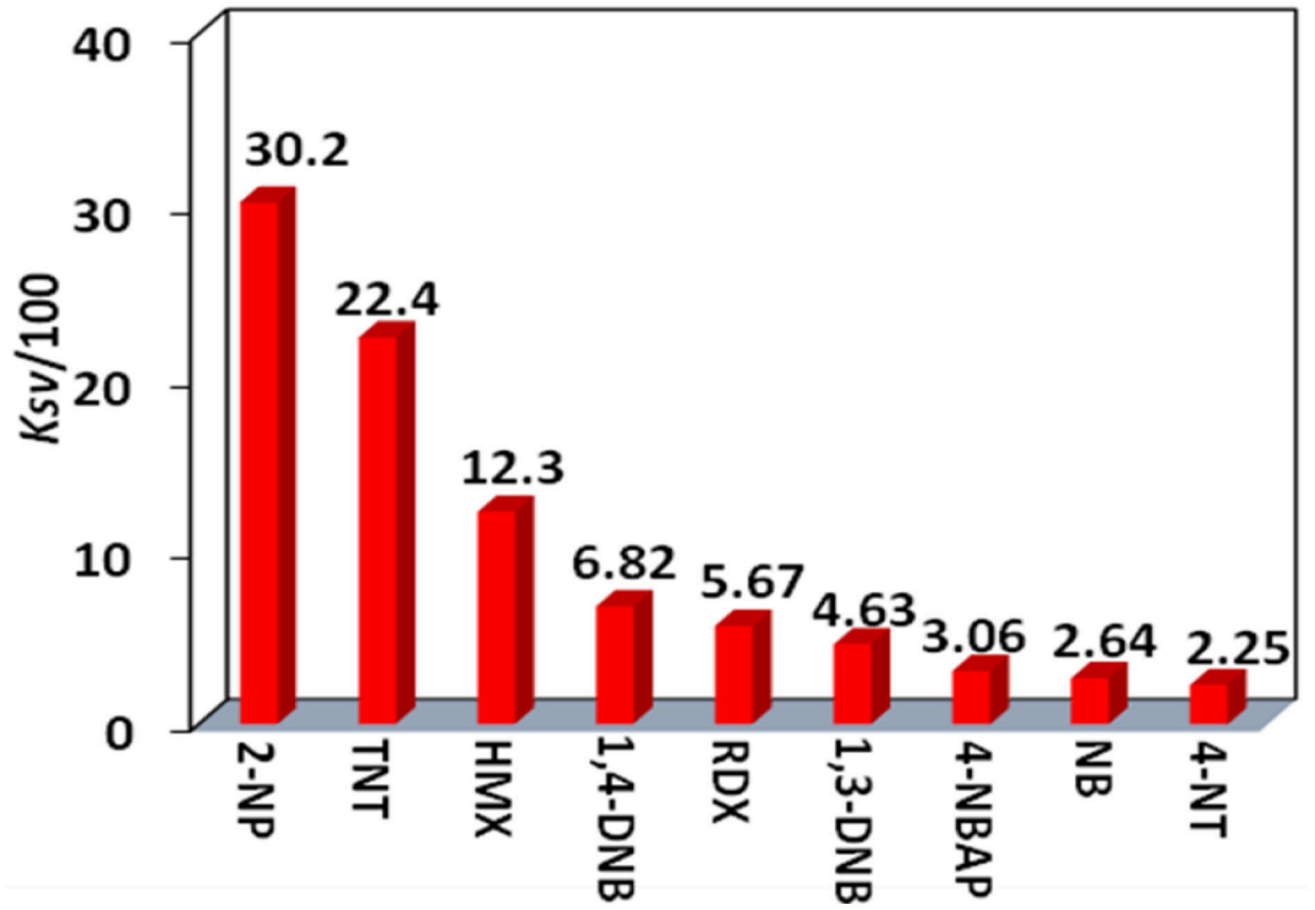
The luminescence enhancement or quenching constants (*K*_*SV*_) of **4** (15 μM) toward nitro explosives.

The Stern-Volmer (SV) equation, *K*_*SV*_ = (*I*_0_/*I*- 1)/[A], can be used to calculate the luminescence enhancement or quenching constants of **4** to the explosives (Xiao et al., 2010). In this equation, *I*_0_ and *I* are the luminescence intensities before and after the addition of the explosive, respectively, and [A] is the molar concentration of the explosive. The *K*_*SV*_ values of **4** to all explosives are shown in Figure 8 (Figure S7, Supporting Information). It was found that **4** shows the highest *K*_*SV*_ value to 2-NP (3,020 M^−1^), indicating that **4** is most sensitive to this explosive. The *K*_*SV*_ values to other explosives are from 225 to 2,240 M^−1^. The luminescence detection limits of **4** to the explosives can be calculated using the 3σ/*K*_*sv*_ equation, where σ is the standard deviation (Qi et al., 2017). The detection limit of **4** to 2-NP is found to be 14.70 μM, indicating that **4** shows high luminescence sensitivity to this explosive at the ppm level.

The perturbation of the added explosives, to the electronic structure of the ligand, may affect the ligand-to-lanthanide energy transfer process in **4**. The luminescent quenching response of lanthanide-based sensors, toward nitroaromatic explosives, can be explained by photoinduced electron transfer (PET) and resonance energy transfer (RET) mechanisms (Li et al., 2013). In both mechanisms, the efficiency of ligand-to-lanthanide energy transfer is an important contributor to the luminescence intensity of the lanthanide complex (María et al., 2017). It was found that the intensities of ligand-centered fluorescence at about 559 nm of **4** are gradually increased with the addition of 2-NP (Figure S8, Supporting Information), indicating that more excitation energy of the Schiff base ligand may be consumed by visible emission. When the concentration of added 2-NP is 400 μM, the NIR emission lifetime and quantum yield of **4** is decreased to 6.42 μs and 0.41%, respectively (Table 1). Thus, the efficiency (η_sens_) of the energy transfer is decreased to 15.95% from 25.0% (without the addition of explosives), demonstrating that the addition of 2-NP may efficiently affect the ligand-to-lanthanide energy transfer process and decreases the luminescence intensity of **4**. The reason for the differences in explosive sensing properties of **4** is more difficult to understand since we do not know the precise nature of the interactions between the complex and the explosives that are introduced. A discussion of the precise nature of these kinds of interactions, as well as the difference between the luminescent response behavior of **2** and **4**, is too speculative to be included in this paper. Our current studies are focused on attempts to isolate and characterize species which may interact with external explosives since this will provide useful information relating to explosive sensing.

## Conclusions

In summary, two types of Cd-Ln complexes **1**–**4** have been successfully synthesized using a new Schiff base ligand (H_2_L), which has a long backbone with two phenyl groups. The length of H_2_L is about 20 Å, which is advantageous for the formation of large metal complexes. **3** and **4** are of nanoscale proportions and their molecular sizes are about 6 × 10 × 15 Å. The long Schiff base ligands show a “twist” configuration in all complexes. The chromogenic Cd/L moieties in **2** and **4** can act as efficient sensitizers to absorb and transfer energy to the Nd^3+^ centers, resulting in typical lanthanide luminescence. The Cd-Nd nanocluster **4** shows NIR luminescent sensing of nitro explosives. The luminescence quenching constant of **4** to 2-NP is 3,020 M^−1^, which is much larger than others (from 225 to 2,240 M^−1^). The detection limit of **4** to 2-NP is 14.70 μM, indicating that **4** has a high sensitivity for this explosive at the ppm level.

## Author Contributions

XY and SH design the Cd-Ln nanoclusters. HC, WJ, DJ, DS, BY, FW and LZ finish the experiment.

### Conflict of Interest Statement

WJ was employed by company Guangzhou Sysmyk New Material Science & Technology Co., Ltd. The remaining authors declare that the research was conducted in the absence of any commercial or financial relationships that could be construed as a potential conflict of interest.

## Supplementary Material

Supporting Information Experimental and characterization details, additional figures and tables, and CIF files (CCDC 1865266-1865269 for 1-4).
