# Supplementary material for: Anion Dependent Self-Assembly of Polynuclear Cd-Ln Schiff Base Nanoclusters: NIR Luminescent Sensing of Nitro Explosives
Source: Front Chem. 2019 Mar 20;7:139. doi: 10.3389/fchem.2019.00139 (PMC6435579; doi:10.3389/fchem.2019.00139)
Supplement: Supporting Information — Experimental and characterization details, additional figures and tables, and CIF files (CCDC 1865266-1865269 for 1-4). [file Table_1.doc]

**Supporting Information**

Anion Dependent Self-assembly of Polynuclear Cd-Ln Schiff Base Nanoclusters: NIR Luminescent Sensing of Nitro Explosives

Hongfen Chen, Xiaoping Yang,* Weizhong Jiang, Dongmei Jiang, Dongliang Shi, Bichen Yuan, Fei Wang, Lijie Zhang and Shaoming Huang*

**Contents**

1. General Procedures...............................................................................................................S2

2. Photophysical Studies...........................................................................................................S2

3. ^1^H NMR spectrum of H_2_L.....................................................................................................S3

4. Powder XRD patterns of **1** and **4**..........................................................................................S4

5. The thermogravimetric analysis of **1**-**4**.................................................................................S5

6. The MS(ESI) spectrum of **1**..................................................................................................S6

7. Photophysical properties of the free ligand H_2_L and **1**-**4**......................................................S7

8. The NIR emission lifetimes of **2** and **4**..................................................................................S8

9. NIR luminescence sensing of **4** towards nitro explosives.............................…….....…...…S9

10. Visible emission response of **4** towards 2-NP..............................................…........…...…S11

11. X-Ray Crystallography........................................................................................................S12

**1. General Procedures**

Metal salts and solvents were purchased from Meryer and used directly without further purification. All reactions were performed under dry oxygen-free dinitrogen atmospheres using standard Schlenk techniques. Physical measurements: Powder XRD: D8ADVANCE; IR: Nicolet IS10 spectrometer. Melting points were obtained in sealed glass capillaries under dinitrogen and are uncorrected. Elemental analyses (C, H, N) were carried out on a EURO EA3000 elemental analysis. Field emission scanning electron microscopy (FESEM) images and EDX spectra were recorded on a Nova NanoSEM 200 scanning electron microscope.

**2. Photophysical Studies**

The UV-visible absorption spectra were recorded at RT using an UV-3600 spectrophotometer. The solvent employed was of HPLC grade. Luminescence spectra in the visible and NIR regions were recorded on a FLS 980 fluorimeter. The light source for excitation and emission spectra was a 450 W xenon arc lamp with continuous spectral distribution from 190 to 2600 nm. Liquid nitrogen cooled Ge PIN diode detector was used to detect the NIR emissions from 800 nm to 1700 nm. The temporal decay curves of the fluorescence signals were stored by using the attached storage digital oscilloscope. The overall emission quantum yields (Φ_em_) were obtained by using an integrating sphere, according to eqn Φ_em_ = *N*_em_ / *N*_abs_, where *N*_em_ and *N*_abs_ are the numbers of emitted and absorbed photons, respectively. The intrinsic quantum yields (Φ_Ln_) of Ln^3+^ emission are calculated using Φ_Ln_ = *τ*/*τ*_0_, where *τ* and *τ*_0_ are the observed emission lifetime and the natural lifetime of Ln^3+^, respectively. Systematic errors have been deducted through the standard instrument corrections. All the measurements were carried out at room temperature.

**3. ^1^H NMR spectrum of H_2_L**


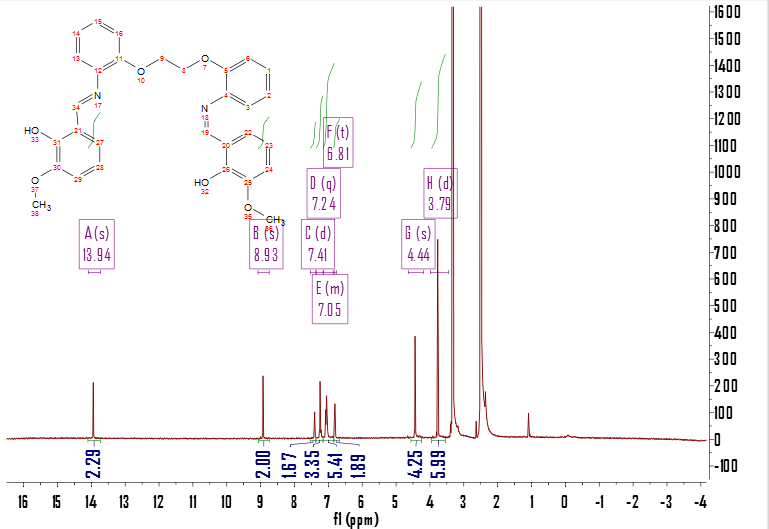


**Figure S1.** ^1^H NMR spectrum of H_2_L in DMSO.

**4. Powder XRD patterns of 1 and 4**


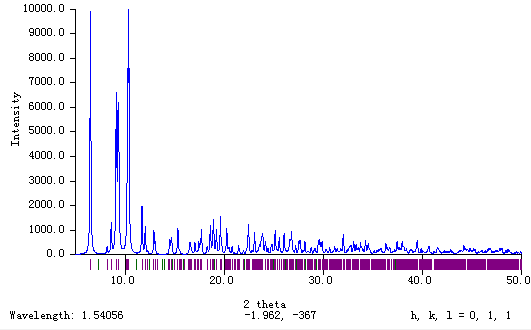

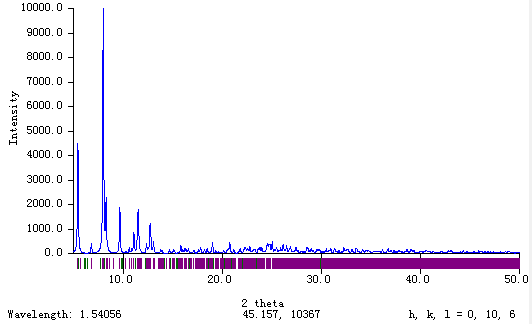


**Figure S2.** Powder XRD patterns of **1** and **4** (the insert pictures were obtained from experiments).

**5. The thermogravimetric analysis of 1-4**

**Figure S3**. The thermogravimetric analysis of **1**-**4**.

**6. The MS(ESI) spectrum of 1**

**Figure S4**. The MS(ESI) spectrum of **1**.

**7. Photophysical properties of the free ligand H_2_L and** **1**-**4**

**Figure S5.** The excitation and visible emission spectra of the free ligand H_2_L and **1**-**4** in CH_3_CN.

**8. The NIR emission lifetimes of 2 and 4**

**Figure S6.** The NIR emission lifetimes of **2** and **4** in CH_3_CN.

**9. NIR luminescent sensing of 4 towards nitro explosives**

**Figure S7.** NIR luminescent sensing of **4** (15 µM) towards nitro explosives in CH_3_CN.

**10. Visible emission response of 4 towards 2-NP**

**Figure S8**. Visible emission response of **4** (15 µM) to the addition of 2-NP in CH_3_CN.

11. X-Ray Crystallography

**Table S1**. Selected bond lengths (Å) and angles (°) for **1**.

La(1)-O(5) 2.442(19)

La(1)-O(2) 2.47(2)

La(1)-O(11) 2.49(2)

La(1)-O(10) 2.57(3)

La(1)-O(7) 2.64(3)

La(1)-O(8) 2.66(3)

La(1)-N(2) 2.70(3)

La(1)-O(3) 2.72(2)

La(1)-N(1) 2.75(2)

La(1)-O(4) 2.83(2)

Cd(1)-O(5) 2.26(2)

Cd(1)-O(2) 2.337(19)

Cd(1)-O(1) 2.46(2)

Cd(1)-Cl(1) 2.470(11)

Cd(1)-Cl(2) 2.472(11)

Cd(1)-O(6) 2.60(2)

O(5)-La(1)-O(2) 68.8(6)

O(5)-La(1)-O(11) 84.5(8)

O(2)-La(1)-O(11) 141.5(8)

O(5)-La(1)-O(10) 79.6(8)

O(2)-La(1)-O(10) 80.5(8)

O(11)-La(1)-O(10) 67.4(9)

O(5)-La(1)-O(7) 89.5(9)

O(2)-La(1)-O(7) 71.6(8)

O(11)-La(1)-O(7) 137.4(9)

O(10)-La(1)-O(7) 152.1(9)

O(5)-La(1)-O(8) 134.0(8)

O(2)-La(1)-O(8) 86.3(7)

O(11)-La(1)-O(8) 131.5(8)

O(10)-La(1)-O(8) 135.2(9)

O(7)-La(1)-O(8) 45.2(9)

O(5)-La(1)-N(2) 67.5(9)

O(2)-La(1)-N(2) 120.0(8)

O(11)-La(1)-N(2) 69.9(9)

O(10)-La(1)-N(2) 127.7(10)

O(7)-La(1)-N(2) 68.9(9)

O(8)-La(1)-N(2) 95.9(9)

O(5)-La(1)-O(3) 154.8(7)

O(2)-La(1)-O(3) 123.5(7)

O(11)-La(1)-O(3) 73.2(7)

O(10)-La(1)-O(3) 81.2(8)

O(7)-La(1)-O(3) 114.8(8)

O(8)-La(1)-O(3) 71.1(8)

N(2)-La(1)-O(3) 113.5(8)

O(5)-La(1)-N(1) 126.5(7)

O(2)-La(1)-N(1) 65.5(7)

O(11)-La(1)-N(1) 116.4(8)

O(10)-La(1)-N(1) 67.0(8)

O(7)-La(1)-N(1) 101.0(8)

O(8)-La(1)-N(1) 68.6(8)

N(2)-La(1)-N(1) 163.8(9)

O(3)-La(1)-N(1) 58.2(7)

O(5)-La(1)-O(4) 123.5(7)

O(2)-La(1)-O(4) 150.3(7)

O(11)-La(1)-O(4) 67.9(8)

O(10)-La(1)-O(4) 126.3(8)

O(7)-La(1)-O(4) 81.1(8)

O(8)-La(1)-O(4) 65.8(8)

N(2)-La(1)-O(4) 57.0(9)

O(3)-La(1)-O(4) 58.4(7)

N(1)-La(1)-O(4) 110.0(7)

O(5)-Cd(1)-O(2) 74.3(7)

O(5)-Cd(1)-O(1) 121.3(8)

O(2)-Cd(1)-O(1) 66.5(7)

O(5)-Cd(1)-Cl(1) 134.9(6)

O(2)-Cd(1)-Cl(1) 98.2(6)

O(1)-Cd(1)-Cl(1) 93.4(6)

O(5)-Cd(1)-Cl(2) 96.1(6)

O(2)-Cd(1)-Cl(2) 136.8(6)

O(1)-Cd(1)-Cl(2) 85.2(6)

Cl(1)-Cd(1)-Cl(2) 116.1(4)

O(5)-Cd(1)-O(6) 64.0(8)

O(2)-Cd(1)-O(6) 122.8(7)

O(1)-Cd(1)-O(6) 170.6(8)

Cl(1)-Cd(1)-O(6) 86.3(7)

Cl(2)-Cd(1)-O(6) 86.5(6)

**Table S2**. Selected bond lengths (Å) and angles (°) for **2**.

Nd(1)-O(2) 2.424(7)

Nd(1)-O(5) 2.434(6)

Nd(1)-O(11) 2.511(7)

Nd(1)-O(10) 2.566(8)

Nd(1)-O(8) 2.605(8)

Nd(1)-O(7) 2.623(8)

Nd(1)-O(3) 2.731(6)

Nd(1)-O(4) 2.740(6)

Nd(1)-N(1) 2.758(8)

Nd(1)-N(2) 2.783(8)

Cd(1)-O(5) 2.284(7)

Cd(1)-O(2) 2.311(8)

Cd(1)-Cl(1) 2.457(3)

Cd(1)-Cl(2) 2.467(3)

Cd(1)-O(1) 2.533(8)

Cd(1)-O(6) 2.595(8)

O(2)-Nd(1)-O(5) 67.5(2)

O(2)-Nd(1)-O(11) 143.0(3)

O(5)-Nd(1)-O(11) 88.8(2)

O(2)-Nd(1)-O(10) 77.6(2)

O(5)-Nd(1)-O(10) 75.1(2)

O(11)-Nd(1)-O(10) 68.7(2)

O(2)-Nd(1)-O(8) 72.1(3)

O(5)-Nd(1)-O(8) 85.3(2)

O(11)-Nd(1)-O(8) 136.2(3)

O(10)-Nd(1)-O(8) 148.5(3)

O(2)-Nd(1)-O(7) 81.3(2)

O(5)-Nd(1)-O(7) 129.6(2)

O(11)-Nd(1)-O(7) 134.4(3)

O(10)-Nd(1)-O(7) 136.3(2)

O(8)-Nd(1)-O(7) 46.8(2)

O(2)-Nd(1)-O(3) 122.6(2)

O(5)-Nd(1)-O(3) 157.7(2)

O(11)-Nd(1)-O(3) 71.8(2)

O(10)-Nd(1)-O(3) 87.4(2)

O(8)-Nd(1)-O(3) 116.2(2)

O(7)-Nd(1)-O(3) 72.6(2)

O(2)-Nd(1)-O(4) 148.9(2)

O(5)-Nd(1)-O(4) 123.8(2)

O(11)-Nd(1)-O(4) 68.0(2)

O(10)-Nd(1)-O(4) 131.8(2)

O(8)-Nd(1)-O(4) 79.7(2)

O(7)-Nd(1)-O(4) 69.7(2)

O(3)-Nd(1)-O(4) 59.58(18)

O(2)-Nd(1)-N(1) 65.3(2)

O(5)-Nd(1)-N(1) 125.2(2)

O(11)-Nd(1)-N(1) 113.9(2)

O(10)-Nd(1)-N(1) 69.3(2)

O(8)-Nd(1)-N(1) 104.6(3)

O(7)-Nd(1)-N(1) 67.2(2)

O(3)-Nd(1)-N(1) 57.6(2)

O(4)-Nd(1)-N(1) 111.0(2)

O(2)-Nd(1)-N(2) 119.3(2)

O(5)-Nd(1)-N(2) 66.2(2)

O(11)-Nd(1)-N(2) 70.8(2)

O(10)-Nd(1)-N(2) 123.5(2)

O(8)-Nd(1)-N(2) 67.2(2)

O(7)-Nd(1)-N(2) 100.2(2)

O(3)-Nd(1)-N(2) 115.2(2)

O(4)-Nd(1)-N(2) 58.0(2)

N(1)-Nd(1)-N(2) 166.5(2)

O(5)-Cd(1)-O(2) 71.9(2)

O(5)-Cd(1)-Cl(1) 104.44(17)

O(2)-Cd(1)-Cl(1) 148.6(2)

O(5)-Cd(1)-Cl(2) 133.95(18)

O(2)-Cd(1)-Cl(2) 92.58(19)

Cl(1)-Cd(1)-Cl(2) 109.64(10)

O(5)-Cd(1)-O(1) 112.2(3)

O(2)-Cd(1)-O(1) 65.4(3)

Cl(1)-Cd(1)-O(1) 88.9(2)

Cl(2)-Cd(1)-O(1) 98.6(2)

O(5)-Cd(1)-O(6) 63.7(2)

O(2)-Cd(1)-O(6) 117.6(2)

Cl(1)-Cd(1)-O(6) 85.7(2)

Cl(2)-Cd(1)-O(6) 88.79(18)

O(1)-Cd(1)-O(6) 172.0(3)

**Table S3**. Selected bond lengths (Å) and angles (°) for **3**.

La(1)-O(2) 2.211(10)

La(1)-O(5) 2.229(10)

La(1)-O(11) 2.238(9)

La(1)-O(8) 2.277(9)

La(1)-O(7) 2.453(10)

La(1)-O(6) 2.575(11)

La(1)-O(12) 2.588(11)

La(1)-O(1) 2.964(12)

La(2)-O(11) 2.363(9)

La(2)-O(8) 2.379(9)

La(2)-O(16) 2.517(13)

La(2)-O(14) 2.527(10)

La(2)-O(17) 2.560(13)

La(2)-O(13) 2.614(11)

La(2)-N(3) 2.657(12)

La(2)-O(9) 2.677(10)

La(2)-O(10) 2.698(10)

La(2)-N(4) 2.721(12)

La(2)-N(6) 2.904(18)

La(2)-N(5) 2.977(13)

Cd(1)-O(19) 2.237(14)

Cd(1)-O(2) 2.282(10)

Cd(1)-O(20) 2.307(13)

Cd(1)-O(5) 2.311(11)

Cd(1)-N(2) 2.372(15)

Cd(1)-N(1) 2.447(14)

O(2)-La(1)-O(5) 71.0(4)

O(2)-La(1)-O(11) 93.8(3)

O(5)-La(1)-O(11) 149.4(4)

O(2)-La(1)-O(8) 147.4(4)

O(5)-La(1)-O(8) 133.7(4)

O(11)-La(1)-O(8) 72.2(3)

O(2)-La(1)-O(7) 103.8(4)

O(5)-La(1)-O(7) 83.1(4)

O(11)-La(1)-O(7) 127.0(3)

O(8)-La(1)-O(7) 66.3(3)

O(2)-La(1)-O(6) 131.2(4)

O(5)-La(1)-O(6) 65.2(4)

O(11)-La(1)-O(6) 113.3(3)

O(8)-La(1)-O(6) 81.1(3)

O(7)-La(1)-O(6) 91.5(4)

O(2)-La(1)-O(12) 81.6(3)

O(5)-La(1)-O(12) 86.3(4)

O(11)-La(1)-O(12) 64.8(3)

O(8)-La(1)-O(12) 116.0(3)

O(7)-La(1)-O(12) 165.7(4)

O(6)-La(1)-O(12) 75.3(4)

O(2)-La(1)-O(1) 58.5(4)

O(5)-La(1)-O(1) 113.3(4)

O(11)-La(1)-O(1) 77.3(3)

O(8)-La(1)-O(1) 89.3(3)

O(7)-La(1)-O(1) 71.0(4)

O(6)-La(1)-O(1) 162.3(4)

O(12)-La(1)-O(1) 122.3(3)

O(11)-La(2)-O(8) 68.3(3)

O(11)-La(2)-O(16) 94.3(4)

O(8)-La(2)-O(16) 79.7(4)

O(11)-La(2)-O(14) 78.7(3)

O(8)-La(2)-O(14) 131.9(3)

O(16)-La(2)-O(14) 138.4(4)

O(11)-La(2)-O(17) 139.0(4)

O(8)-La(2)-O(17) 84.1(4)

O(16)-La(2)-O(17) 49.8(4)

O(14)-La(2)-O(17) 140.2(4)

O(11)-La(2)-O(13) 73.2(3)

O(8)-La(2)-O(13) 87.3(3)

O(16)-La(2)-O(13) 164.7(4)

O(14)-La(2)-O(13) 49.1(3)

O(17)-La(2)-O(13) 137.1(4)

O(11)-La(2)-N(3) 121.5(3)

O(8)-La(2)-N(3) 68.0(3)

O(16)-La(2)-N(3) 113.7(4)

O(14)-La(2)-N(3) 104.5(3)

O(17)-La(2)-N(3) 70.0(4)

O(13)-La(2)-N(3) 67.8(4)

O(11)-La(2)-O(9) 147.9(3)

O(8)-La(2)-O(9) 126.2(3)

O(16)-La(2)-O(9) 115.5(4)

O(14)-La(2)-O(9) 71.2(3)

O(17)-La(2)-O(9) 73.0(4)

O(13)-La(2)-O(9) 78.8(3)

N(3)-La(2)-O(9) 58.5(3)

O(11)-La(2)-O(10) 122.6(3)

O(8)-La(2)-O(10) 152.2(3)

O(16)-La(2)-O(10) 74.3(4)

O(14)-La(2)-O(10) 75.5(3)

O(17)-La(2)-O(10) 71.9(4)

O(13)-La(2)-O(10) 119.7(4)

N(3)-La(2)-O(10) 114.2(3)

O(9)-La(2)-O(10) 60.1(3)

O(11)-La(2)-N(4) 66.7(3)

O(8)-La(2)-N(4) 121.2(4)

O(16)-La(2)-N(4) 68.3(4)

O(14)-La(2)-N(4) 71.4(4)

O(17)-La(2)-N(4) 107.4(4)

O(13)-La(2)-N(4) 112.9(4)

N(3)-La(2)-N(4) 170.5(4)

O(9)-La(2)-N(4) 112.0(3)

O(10)-La(2)-N(4) 56.7(4)

O(11)-La(2)-N(6) 116.7(5)

O(8)-La(2)-N(6) 81.3(4)

O(16)-La(2)-N(6) 24.2(5)

O(14)-La(2)-N(6) 146.3(4)

O(17)-La(2)-N(6) 25.6(5)

O(13)-La(2)-N(6) 160.1(5)

N(3)-La(2)-N(6) 92.7(5)

O(9)-La(2)-N(6) 94.8(5)

O(10)-La(2)-N(6) 71.0(4)

N(4)-La(2)-N(6) 87.0(5)

O(11)-La(2)-N(5) 73.6(3)

O(8)-La(2)-N(5) 109.4(3)

O(16)-La(2)-N(5) 159.8(4)

O(14)-La(2)-N(5) 24.8(3)

O(17)-La(2)-N(5) 146.5(4)

O(13)-La(2)-N(5) 24.4(3)

N(3)-La(2)-N(5) 86.5(3)

O(9)-La(2)-N(5) 74.5(3)

O(10)-La(2)-N(5) 98.3(4)

N(4)-La(2)-N(5) 91.8(4)

N(6)-La(2)-N(5) 167.9(5)

O(19)-Cd(1)-O(2) 83.1(4)

O(19)-Cd(1)-O(20) 148.1(5)

O(2)-Cd(1)-O(20) 126.2(4)

O(19)-Cd(1)-O(5) 125.6(5)

O(2)-Cd(1)-O(5) 68.3(3)

O(20)-Cd(1)-O(5) 81.6(4)

O(19)-Cd(1)-N(2) 83.5(5)

O(2)-Cd(1)-N(2) 121.0(4)

O(20)-Cd(1)-N(2) 89.7(5)

O(5)-Cd(1)-N(2) 74.4(4)

O(19)-Cd(1)-N(1) 90.7(5)

O(2)-Cd(1)-N(1) 74.0(4)

O(20)-Cd(1)-N(1) 86.7(5)

O(5)-Cd(1)-N(1) 121.6(4)

N(2)-Cd(1)-N(1) 162.8(5)

**Table S4**. Selected bond lengths (Å) and angles (°) for **4**.

Nd(1)-O(2) 2.218(10)

Nd(1)-O(5) 2.241(12)

Nd(1)-O(11) 2.271(10)

Nd(1)-O(8) 2.292(10)

Nd(1)-O(7) 2.444(11)

Nd(1)-O(6) 2.584(12)

Nd(1)-O(12) 2.609(11)

Nd(1)-O(1) 2.888(13)

Nd(2)-O(11) 2.397(10)

Nd(2)-O(8) 2.414(10)

Nd(2)-O(16) 2.574(15)

Nd(2)-O(14) 2.579(11)

Nd(2)-O(17) 2.612(14)

Nd(2)-O(13) 2.654(12)

Nd(2)-N(3) 2.699(14)

Nd(2)-O(9) 2.700(11)

Nd(2)-O(10) 2.709(11)

Nd(2)-N(4) 2.749(13)

Nd(2)-N(6) 2.95(2)

Cd(1)-O(19) 2.262(16)

Cd(1)-O(2) 2.265(11)

Cd(1)-O(5) 2.301(12)

Cd(1)-O(20) 2.322(15)

Cd(1)-N(2) 2.348(17)

Cd(1)-N(1) 2.440(15)

O(2)-Nd(1)-O(5) 69.8(4)

O(2)-Nd(1)-O(11) 93.5(4)

O(5)-Nd(1)-O(11) 148.4(4)

O(2)-Nd(1)-O(8) 148.8(4)

O(5)-Nd(1)-O(8) 134.0(4)

O(11)-Nd(1)-O(8) 72.9(3)

O(2)-Nd(1)-O(7) 104.5(4)

O(5)-Nd(1)-O(7) 82.9(4)

O(11)-Nd(1)-O(7) 128.2(4)

O(8)-Nd(1)-O(7) 66.7(4)

O(2)-Nd(1)-O(6) 130.3(4)

O(5)-Nd(1)-O(6) 65.3(4)

O(11)-Nd(1)-O(6) 114.0(4)

O(8)-Nd(1)-O(6) 80.7(4)

O(7)-Nd(1)-O(6) 90.3(4)

O(2)-Nd(1)-O(12) 81.2(4)

O(5)-Nd(1)-O(12) 86.6(4)

O(11)-Nd(1)-O(12) 63.9(4)

O(8)-Nd(1)-O(12) 115.1(3)

O(7)-Nd(1)-O(12) 165.3(4)

O(6)-Nd(1)-O(12) 75.9(4)

O(2)-Nd(1)-O(1) 59.7(4)

O(5)-Nd(1)-O(1) 114.0(4)

O(11)-Nd(1)-O(1) 76.1(4)

O(8)-Nd(1)-O(1) 89.5(4)

O(7)-Nd(1)-O(1) 72.5(4)

O(6)-Nd(1)-O(1) 162.6(4)

O(12)-Nd(1)-O(1) 121.4(4)

O(11)-Nd(2)-O(8) 68.6(3)

O(11)-Nd(2)-O(16) 97.0(4)

O(8)-Nd(2)-O(16) 82.1(4)

O(11)-Nd(2)-O(14) 78.1(3)

O(8)-Nd(2)-O(14) 130.0(4)

O(16)-Nd(2)-O(14) 139.5(4)

O(11)-Nd(2)-O(17) 141.7(5)

O(8)-Nd(2)-O(17) 87.8(4)

O(16)-Nd(2)-O(17) 48.5(5)

O(14)-Nd(2)-O(17) 137.7(5)

O(11)-Nd(2)-O(13) 73.7(4)

O(8)-Nd(2)-O(13) 86.7(4)

O(16)-Nd(2)-O(13) 167.6(4)

O(14)-Nd(2)-O(13) 47.8(4)

O(17)-Nd(2)-O(13) 136.8(5)

O(11)-Nd(2)-N(3) 121.8(4)

O(8)-Nd(2)-N(3) 67.5(4)

O(16)-Nd(2)-N(3) 112.5(5)

O(14)-Nd(2)-N(3) 103.6(4)

O(17)-Nd(2)-N(3) 70.7(5)

O(13)-Nd(2)-N(3) 67.6(4)

O(11)-Nd(2)-O(9) 147.8(3)

O(8)-Nd(2)-O(9) 125.4(3)

O(16)-Nd(2)-O(9) 112.9(4)

O(14)-Nd(2)-O(9) 71.4(4)

O(17)-Nd(2)-O(9) 70.6(5)

O(13)-Nd(2)-O(9) 78.1(4)

N(3)-Nd(2)-O(9) 58.1(4)

O(11)-Nd(2)-O(10) 121.5(4)

O(8)-Nd(2)-O(10) 155.2(4)

O(16)-Nd(2)-O(10) 74.4(4)

O(14)-Nd(2)-O(10) 74.5(4)

O(17)-Nd(2)-O(10) 70.9(4)

O(13)-Nd(2)-O(10) 117.3(4)

N(3)-Nd(2)-O(10) 114.4(4)

O(9)-Nd(2)-O(10) 59.9(4)

O(11)-Nd(2)-N(4) 66.0(4)

O(8)-Nd(2)-N(4) 122.2(4)

O(16)-Nd(2)-N(4) 70.0(4)

O(14)-Nd(2)-N(4) 71.4(4)

O(17)-Nd(2)-N(4) 107.1(5)

O(13)-Nd(2)-N(4) 112.1(4)

N(3)-Nd(2)-N(4) 170.2(4)

O(9)-Nd(2)-N(4) 112.1(4)

O(10)-Nd(2)-N(4) 56.5(4)

O(11)-Nd(2)-N(6) 119.1(6)

O(8)-Nd(2)-N(6) 83.8(5)

O(16)-Nd(2)-N(6) 23.7(5)

O(14)-Nd(2)-N(6) 146.0(5)

O(17)-Nd(2)-N(6) 24.8(5)

O(13)-Nd(2)-N(6) 159.4(6)

N(3)-Nd(2)-N(6) 91.8(6)

O(9)-Nd(2)-N(6) 92.5(5)

O(10)-Nd(2)-N(6) 71.5(5)

N(4)-Nd(2)-N(6) 88.5(6)

O(19)-Cd(1)-O(2) 82.7(5)

O(19)-Cd(1)-O(5) 124.9(5)

O(2)-Cd(1)-O(5) 68.0(4)

O(19)-Cd(1)-O(20) 148.7(6)

O(2)-Cd(1)-O(20) 125.8(5)

O(5)-Cd(1)-O(20) 82.2(5)

O(19)-Cd(1)-N(2) 83.6(6)

O(2)-Cd(1)-N(2) 120.9(5)

O(5)-Cd(1)-N(2) 74.2(5)

O(20)-Cd(1)-N(2) 90.4(6)

O(19)-Cd(1)-N(1) 91.1(5)

O(2)-Cd(1)-N(1) 74.0(4)

O(5)-Cd(1)-N(1) 121.3(4)

O(20)-Cd(1)-N(1) 85.8(5)

N(2)-Cd(1)-N(1) 163.2(5)
